# What matters in white matter dementia?

**DOI:** 10.1590/s1980-57642008dn10200004

**Published:** 2007

**Authors:** Leonardo Caixeta

**Affiliations:** 1 MD, PhD, Adjunct Professor of Neuroscience, Federal University of Goiás (UFG). Coordinator, Cognitive and Behavioral Neurology Unit, Hospital das Clínicas - UFG.

**Keywords:** dementia, white matter, neuropsychology, psychiatric symptoms

## Abstract

Dementia studies has primarily focused on disorders of the cerebral cortex and subcortical gray matter, what originated the concepts of cortical and subcortical dementias respectively. Dementia related mainly with cerebral white matter have received less attention. We present five different cases, each one illustrative of a dementia subtype that could be assigned under the category of ‘white matter dementia’: CADASIL, progressive subcortical gliosis, progressive multifocal leucoencephalopathy, normopressure hydrocephalus and brain injury. Besides that, recent clinical and scientific literature on white matter dementia was reviewed. The composition of exuberant psychiatric symptoms and personality changes (mainly apathy, but also desinhibition) with neurological signs (pyramidal alone or associated with extrapyramidal signs, ataxia and urinary incontinence) and with specific cognitive impairment (mentioned above), should rise strongly the possibility of a white-matter dementia, instead of a cortical or subcortical form of dementia.

## Abstract (pt)

Os trabalhos na área de demência têm enfocado primordialmente os distúrbios do córtex cerebral e da substância cinzenta subcortical, o que originou os conceitos de demência cortical e subcortical, respectivamente. A demência relacionada sobretudo com a substância branca tem recebido menor atenção. Apresentamos cinco casos ilustrativos de subtipos que pudessem ser considerados na categoria de ‘demência da substância branca’: CADASIL, gliose subcortical progressiva, leucoencefalopatia multifocal progressiva, hidrocefalia de pressão normal e traumatismo craniano. Além disso, conduzimos uma revisão da literatura científica atual sobre demência da substância branca. A composição de sintomas psiquiátricos exuberantes e mudanças de personalidade (sobretudo apatia, mas também desinibição) com sinais neurológicos (sinais piramidais isolados ou em associação com sinais extrapiramidais, ataxia e incontinência urinária) e com prejuízo cognitivo específico (mencionados acima), deve levantar fortemente a possibilidade de uma demência da substância branca, ao invés de uma forma de demência do tipo cortical ou subcortical.

The study of higher function in humans requires consideration of all the neural tissues in the brain. Long neglected as a contributor to the organization of cognitive and emotional operations, cerebral white matter is now the subject of substantial effort toward improving understanding in this area. Among the many approaches that can address this area usefully, the study of individuals with white matter disorders offers a wealth of clinical insights that exploit the time-tested lesion method of behavioral neurology.^1^

White matter comprises nearly half brain volume and plays a key role in development, aging, and many neurologic and psychiatric disorders during life span. More than 100 disorders exist in which white matter neuropathology is the primary, or a prominent feature.^2^ A variety of neurobehavioral syndromes may stem from these disorders.

Recent years have witnessed significant advances in these disorders. Imaging techniques namely computerized tomography (CT) and especially magnetic resonance (MR) imaging reveal changes in CNS white matter. The distribution and characteristics of the changes in white matter help to distinguish different varieties of white matter diseases. Newer techniques like MR spectroscopy also identify the underlying biochemical changes in some of these disorders. Electrophysiological procedures such as electromyo­graphy and nerve conduction studies help to detect involvement (even sub-clinical) of the peripheral nervous system. Detection of the underlying biochemical and enzymatic defects has been possible in some conditions. Identification of the genetic defect has further helped genetic counseling and prenatal diagnosis.^2^

Dementia studies have primarily focused on disorders of the cerebral cortex and subcortical gray matter, giving rise to the concepts of cortical and subcortical dementias respectively. Dementias related mainly to cerebral white matter have received less attention. White matter dementia is a term introduced by Filley in 1988 to call attention to the morbidity caused by disabling cognitive loss in patients who have white matter disorders.^3^ Since then, white matter dementia has been proposed as a clinical entity.^1-6^ The extensive body of accumulated knowledge on white matter outlined earlier has enabled the concept of white matter dementia to become better established and more accepted by the scientific community.

As this article cannot be encyclopedic, it will be necessarily restricted to more prevalent or illustrative etiologies of white matter dementia in adults. We present five different cases, each illustrative of a dementia subtype that can be classified under the category of ‘white matter dementia’, namely: CADASIL, progressive subcortical gliosis, progressive multifocal leucoencephalopathy, normopressure hydrocephalus and brain injury. In addition, a brief review has been conducted in order to compile a list of the main white matter disorders associated with dementia or cognitive impairment.

## Case reports

### Case 1 (probable CADASIL)

A 62 year-old white physician began, 10 years earlier, to develop frequent mood disorder (emotional lability, dysphoria, distress) that was treated as depression. He also presented with personality changes: he used to leave his home and abandon his wife with no clear reason or because of irrelevant reasons for long periods, during which time he would settle in at a friend’s house; when he decided to return, he appeared looking like a beggar, dirty and ragged; sometimes he acted as a prodigal man, making donations of his belongings in an inappropriate manner or doing business with evident detriment to himself; attitudes denoting bad character with constant lies and antisocial behavior, in contrast to his strict moral education. Also, he presented delusional ideas (jealous delusions where he would beat his wife and threaten her with knives). He showed no awareness of his behavior alterations.

His score on the mini-mental state examination at this time was 19. A more comprehensive neuropsychological evaluation showed severe executive functions deficits, memory impairment characterized by difficulties in declarative memory, retrograde and mainly anterograde, severe temporal disorientation as well as difficulties in visuospatial tests. On the *Hooper test*, his score was 11/30 and his errors were related with organization of the visual data, but not associated with visual recognition. He did not perform (failed) on cubes and codes (*WAIS*). *His digit span forward* was normal, but backwards was impaired (percentile .01), demonstrating working memory impairment. On the *Mattis scale*, he scored 93 points while his performance was impaired mainly in executive functions (conceptual formation, working memory, difficulties with new settings).

Mid-stage in his evolution he presented with parkinsonian features: *petit pas* gait, rest tremor, rigidity and bradikinesia. He evolved with progressive apathy and lack of initiative, insight impairment, reduction of verbal output until reaching complete mutism, culminating in a fully demented state (CDR 3), becoming restricted to his room, with frequent choking problems and completely dependent on caregiver attention.

Diagnosis of CADASIL was made based on his clinical features: beginning with migraine and behavior alteration many years before dementia onset, associated with cerebrovascular insults without a logical explanation (absence of cardiovascular risks) in a dominant inheritance pattern (currently, 40 members of his family have the same clinical picture, outcome and neuroimaging features). The search for notch 3 mutations in order to confirm the diagnosis is in progress.

**Figure 1:**
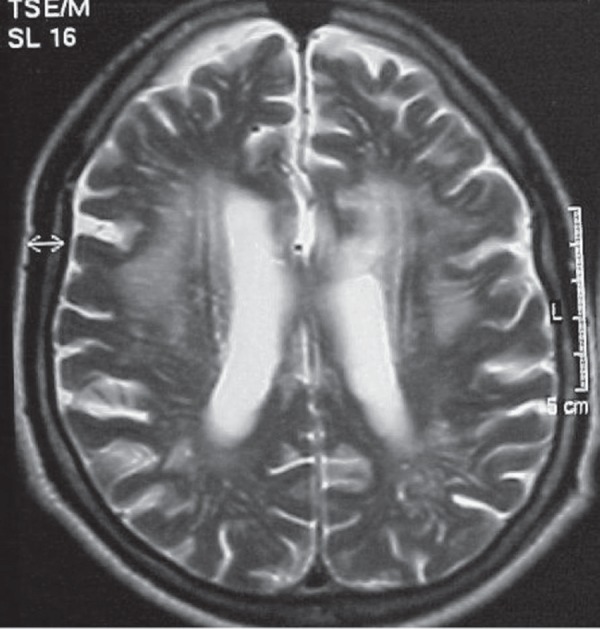
Case 1 – RM (T2 axial) showing multiple confluent white matter lesions, not reaching the “U” fibers.

The results from his behavior evaluation, as assessed by NPI, are summarized in Table 2 and his MRI is presented in Figure 1.

**Table 2:** Neuropsychiatric Inventory (NPI) of the fi ve cases.

Domain	Case 1 (FxS=T)	Case 2 (FxS=T)	Case 3 (FxS=T)	Case 4 (FxS=T)	Case 5 (FxS=T)
Delusions	4x3=12	1x3=3	0	3x3=9	4x3=12
Hallucination	3x3=9	0	0	3x3=9	0
Agitation/aggression	1x3=3	4x3=12	3x3=9	0	4x3=12
Depression/Dysphoria	1x2=2	0	3x3=9	1x1=1	2x3=6
Anxiety	1x1=1	0	3x2=6	0	4x3=12
Euphoria/elation	0	0	0	0	4x3=12
Apathy/indifference	4x3=12	4x1=4	4x1=4	3x2=6	2x2=4
Disinhibition	4x3=12	4x3=12	3x3=9	0	4x3=12
Irritability/lability	4x3=12	1x1=1	3x2=6	1x1=1	4x3=12
Aberrant motor behavior	3x1=3	4x3=12	4x3=12	3x2=6	2x3=6
Nighttime behaviors	4x3=12	0	3x2=6	0	0
Appetite/eating	3x1=3	3x2=6	4x2=8	0	0
Accumulated Total	81	50	69	27	88

F, frequency; S, severity; T, total.

### Case 2 (probable progressive subcortical gliosis)

A 58 year-old white woman commenced symptoms with an erotomanic episode: she fell in love in a delusional fashion with a family friend and became convinced of the reciprocity of these feelings simply because this man visited her home once (to meet her father) and, when bidding farewell gave her a kiss on the cheek (as he did to her sisters). Six months on, when a highly respected nephew was murdered, she reacted in an inappropriate manner to the situation (according to her family), intending to get a gun in order to murder the assassin. Paradoxically, however, some days later, she did not mention her nephew further, becoming indifferent to the fact. In this period, she started wandering and became lost, remaining away for two days until found by a relative. Her family became certain that something serious was happening to her personality, since she was progressively behaving inadequately, wastefully (used to give her belongings to strangers) while at the same time egocentric (without demonstrating involvement with the surrounding relatives, their problems, their lives), self-neglected and indifferent to her domestic duties, without notion of danger (used to climb up to high places and then did not know how to get down), being unaware of her condition, and increasingly requiring caregiver supervision.

Approximately one year after onset of the disease, she began to suffer from urinary incontinence, an early sign of the process. She steadily evolved with progressive apathy and lack of initiative, reduction of verbal output until reaching complete mutism, culminating in a fully demented state (CDR 3), becoming room-bound and completely dependent on caregiver attention. Four years after her disease commenced, her MMSE score was 20.

A more comprehensive neuropsychological evaluation showed additional data. Her attention span was relatively low. In the copy of alternated symbols she rotated part of drawings, lost sequence, the patient used previously established stereotypes, modified symbols by letters, compounding words (ex. ΠΛΠΛ=MArIA) as well as perseverations (alternated drawings). On digit repetition, she was not able to follow instructions and did an addition with the numbers presented, compromising performance (*digit span forward - WAIS-R*). Mental control was also impaired: she tended to calculate by addition, instead of repeating the reported digits backwards (*digit span backwards* - WAIS-R). She performed badly on verbal fluency, with perseveration, slow verbal output, as well as being unable to abandon an answer which was initially correct (*verbal fluency test for animals and person names*) and then incorrect on next evaluation (FAS). Attentional deficits also became evident when demands for inhibitory control were elicited. Her failings in this area were evident by the following performance: slow production, significant loss of setting, incomplete visual screening of stimuli, inability to make a sequence of numbers and letters (*Trail Making Test*), difficulty of giving an unusual answer instead of a usual one, slow performance and irregular difficulty in naming colors (not in all answers) (*Stroop Test*). Flexibility was very impaired. The patient did not benefit from external stimulation to guide her performance and as a consequence, she perseverated in wrong answers, did not contextualize her actions and showed inability in forming abstract concepts (*Wiscosin Card Sorting Test*). In motor tests, difficulties were noted with reproduction of manual sequences, even in the presence of a model, as well as in coordination of alternated patterns (*Finger and Palm Motor Series and Ozeretsky*). Constructive praxia was impaired, as in the copy of simple figures, for complex, bi and tri-dimensionals (prompted drawing, drawn reproductions - *Strub Blac*- and *Rey I Figure*). Naming was deficient and patient did not benefit from categorical prompts to answer correctly, nor was able to significantly recognize figures after phonemic prompts (*Boston Naming Test*). Verbal fluency was also reduced, as for larger categories (animals and people names) and more restricted categories (FAS). She had average performance on verbal and visual memory tests, immediate or delayed. Additionally, perseverations occurred. The quantitative results allied to the quality of answers indicated bilateral frontal impairment, as well as fronto-basal involvement.

Her diagnosis was based on a clinical picture associated with evolutive MRI evaluation that showed the classical feature of progressive bilateral white matter gliosis, restricted to frontal lobes, even in the latter stages of the disease.

**Figure 2:**
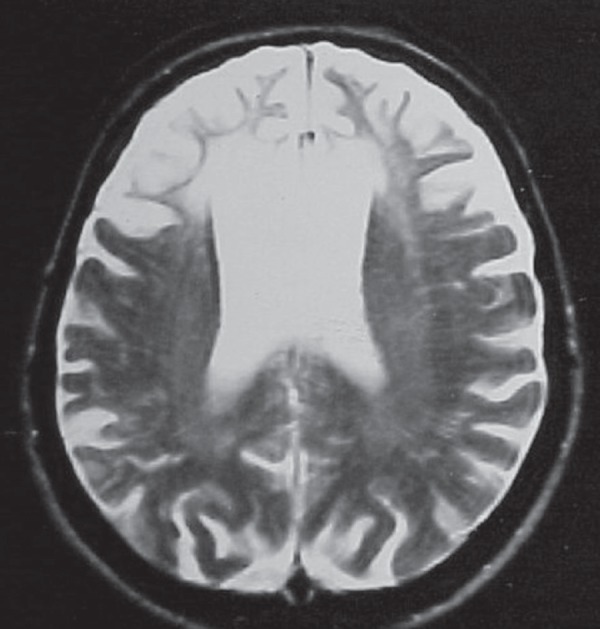
Case 2 – RM (T2 axial) showing bilateral white matter hypointense signal, sug gestive of gliosis, restricted to frontal lobes.

The result of her behavior evaluation as assessed by NPI is summarized in Table 2. Her MRI is presented in Figure 2.

### Case 3 (progressive multifocal leukoencephalopathy- PML)

A 38 year-old white man, HIV positive, had presented in the last four months with progressive and cumulative neurological focal signs: motor deficits and Babinski reflex on the right side of the body, along with expression dysphasia. He also presented at times with somnolence, lassitude, but also agitation and aberrant motor behavior. Other cognitive deficits observed using an ecological neuropsychological approach included: general slowing of cognition, disexecutive syndrome (concretism, impaired conceptualization, no insight, planning difficulties, without social intelligence), reduced verbal output and word fluency, expressive and comprehensive dysphasia (he was only capable of obeying simple commands), severe attention deficit (including impaired eye-to-eye contact), amnesia, temporal disorientation. His spatial orientation within the infirmary was largely normal. Formal neuropsychological assessment proved impossible because of mental confusion and expressive dysphasia. He died at CDR 3, five months after onset of symptoms.

**Figure 3:**
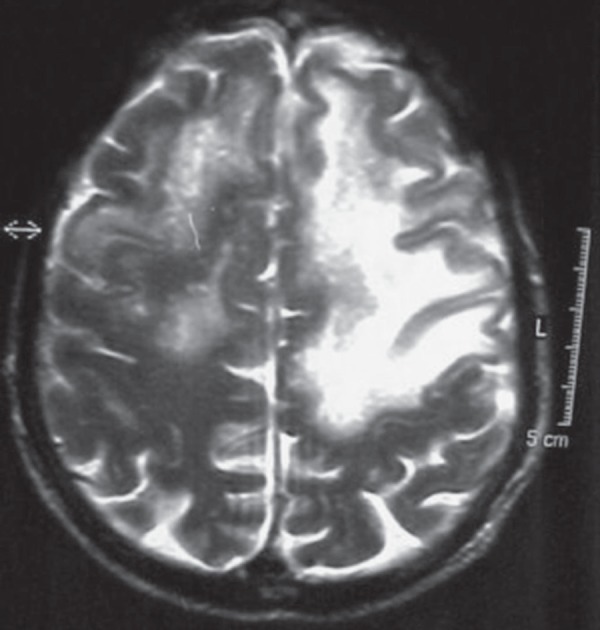
Case 3 – RM (T2 axial) showing mul tiple confluent white matter lesions, mainly on the left, reaching “U” fibers.

The result of his behavior evaluation according to NPI is summarized in Table 2. His MRI is presented in Figure 3.

### Case 4 (normal pressure hydrocephalus)

An 80 year-old Japanese man presented with a history of emotional lability associated with gait difficulties that fluctuated in intensity throughout the day, suffering frequent falls, occasional urinary incontinence, memory impairment (he used to forget where he had placed objects, as well as meetings, difficulty in recalling overlearned material), temporal disorientation, impaired speed of information processing, visuomotor difficulties, reduced verbal fluency, auditive hallucinations, wandering, misidentification syndrome. His score on the mini-mental state examination was 23 before ventriculoperitoneal shunt and 28 after the successful surgical procedure. A more comprehensive neuropsychological evaluation before the shunt showed severe executive dysfunction (conceptualization, abstraction, working memory, social tactile), reduced word fluency (seven animals in one minute), sustained attention deficit, *Boston Naming Test* within normal percentile, without agnosias. In the clock drawing test, he scored just two points, but did not show any other marked praxic impairment. He showed moderate memory impairment (difficulties in immediate memory, retention and recognition). In general, all the cognitive processes showed impaired speed of information processing. He had no insight of his deficits, particularly behavior disorders. The result of his behavior evaluation as assessed by NPI is summarized in Table 2.

His MRI is presented in Figure 3.

### Case 5 (brain injury)

A 26-year-old, white male was involved in a severe car crash seven years prior, in which he presented cranial traumatism, loss of awareness and was kept as an patient in the intensive care unit for two weeks in coma. During hospital stay, he presented with slight motor deficits (a mild left hemiparesis). After his discharge from the hospital, severe behavior and personality changes gradually became apparent. He became impulsive, aggressive, disinhibited (moria), without compassion even involving vulnerable individuals (pseudopsychopathy). He presented obsessive-compulsive symptoms (checker type) and began to present a misidentification syndrome in which he identified familiar people (parents, brothers, his doctor) as being doubles (Capgras delusion). His severe executive dysfunction impaired him in day-to-day activities, making him dependent on caregivers in some functions (hygiene, social behavior, decision making) and inadequate in social relationships (manifesting no insight). His MMSE score was 19 (he had 11 years of schooling). He had a severe impairment in frontal executive tests (*Stroop, Wisconsin Carding Sort Test*, conceptual formation and reasoning - proverbs, similarities). Sustained attention was also severely impaired (shown by cancellation tests). Memory deficits (difficulties in immediate memory, retention and recognition) were observed in RAVTL. He performed badly on *trail making A and B, Mazes* and other visuospatial tests (copy of a cube and other tridimensional figures). His performance on the *Boston Naming Test* was normal, as well as in the cookie theft picture and other language and spatial orientation tests.

**Figure 5:**
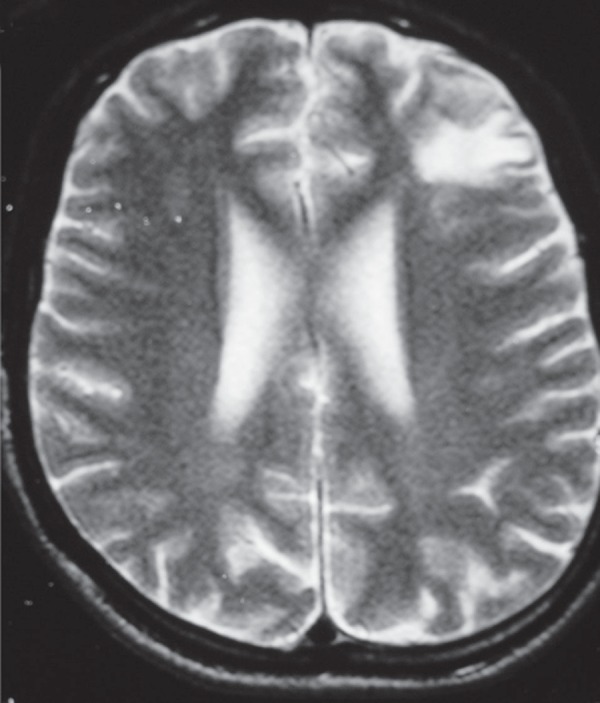
Case 5 – RM (T2 axial) showing a subcortical white matter lesion in the right frontal lobe.

The result of his behavior evaluation according to the NPI is summarized in Table 2. His MRI is presented in Figure 5.

## Discussion

From a nosological perspective, several different causes of dementia and cognitive impairment may be related to white matter pathology, according to our brief review.^6-22^ These are outlined in a summarized form in Table 1.

**Table 1:** Causes of cognitive impairment and dementia related to white matter pathology.

**Vascular diseases**^6-8^	**Demyelinative diseases**^9, 10^
Binswanger's disease	Multiple sclerosis
Cerebral autosomal dominant arteriopathy with subcortical infarcts	Acute disseminated encephalopathy
Leukoencephalopathy	Acute hemorrhagic leukoencephalopathy
Leukoaraiosis	Schilder's disease
Cerebral amyloid angiopathy	Marburg's disease
White matter disease of prematurity	Balò's concentric sclerosis
Migraine	**Infl ammatory diseases^15, 16^**
**Infectious diseases^11-14^**	Systemic lupus erythematosus
Acquired immunodeficiency syndrome dementia complex	Behçet's disease
Progressive multifocal leukoencephalopathy	Sjögre's syndrome
Subacute sclerosing panencephalitis	Wegener's granulomatosis
Progressive rubella panencephalitis	Temporal arteritis
Varicella zoster encephalitis	Polyarteritis nodosa
Cytomegalovirus encephalitis	Scleroderma
Lyme encephalopathy	Isolated angiitis of the central nervous system
**Hydrocephalus^17^**	Sarcoidosis
Early hydrocephalus	**Toxic leukoencephalopathies^18^**
Hydrocephalus ex vacuo	Cranial irradiation
Normal pressure hydrocephalus	Therapeutic drugs
**Genetic disorders^19^**	Drugs of abuse
Leukodystrophies	Environmental toxins
Aminoacidurias	**Metabolic disorders^20^**
Phakomatoses	Cobalamin defi ciency
Mucopolysaccharidoses	Folate defi ciency
Muscular dystrophy	Central pontine myelinolysis
Callosal agenesis	Hypoxia
**Traumatic disorders^21^**	Hypertensive encephalopathy
Traumatic brain injury	Eclampsia
Shaken baby syndrome	High altitude cerebral edema
Corpus callosotomy	
**Neoplasms^22^**	
Gliomatosis cerebri	
Diffusely infi ltrative gliomas	
Primary cerebral lymphoma	
Focal white matter tumors	

Each of our reported cases is representative of a major cause of white matter dementia: vascular (CADASIL), primary degenerative (progressive subcortical gliosis), infectious (progressive multifocal leukoencephalopathy), hydrocefalic and traumatic.

From a syndromic perspective, a wide range of focal neurobehavioral syndromes and psychiatric disorders can be related to dysfunction of myelinated tracts. In 1965, Norman Geschwind^23^ proposed the notion of cerebral disconnection as a mechanism of neurobehavioral dysfunction, thus strongly suggesting the importance of white matter lesions in brain-behavior relationships. At the turn of the twenty-first century, the notion of distributed neural networks as championed by M-Marcel Mesulan^24^ had become widely accepted, further suggesting that white matter plays a central role in the elaboration of human behavior.^25^

The clinical scenario of white matter diseases is usually non-specific and many affected patients can present a primary psychiatric disorder, and indeed, in retrospect, many patients who have white matter dementia have had early psychiatric dysfunction that preceded measurable cognitive impairment.^5^ Indeed, cases 1 and 2 presented initially with psychiatric symptoms when no cognitive impairment was perceivable. Their white matter lesions included mainly frontal lobes, were associated with marked delusions (erotic in case 2, paranoid in case 1, 2 and 4, Capgras delusion in case 4) and personality changes (in all cases). Personality changes observed in our cases, in which a progressive degenerative process was underway (1 and 2), commenced presentation with disinhibition as the main personality feature which later (some years later) transformed to apathy. This may reflect the progression and extent of the white matter damage process, leading to a “frontal leucothomy” that culminates in profound apathy. Literature has reported that dysfunction of the frontal cortex associated with white-matter lesions appears to contribute to the clinical picture of some cases of psychosis, mainly late-life variants.^26,27^ White matter diseases presenting with psychosis can yield important insights into the neurobiology of psychosis. Less significant evidence has associated white matter lesions, mainly frontal and temporal, with personality changes.^28,29^

In general, patients with white matter dementia scored high on the NPI irrespectively of the subjacent cause of their dementia. Some domains scored higher than others in this group and this may be explained by particular expression of psychopathology related with white matter disease. Domains of delusion, agitation, apathy, disinhibition and aberrant motor behavior scored higher, in general, than elation, hallucinations, depression and nighttime behavior. Further study on a larger number of patients, along with statistical analysis is required to confirm this notion.

White matter dementia can be linked to a particular profile, characterized by sustained attention deficit, executive dysfunction, memory retrieval deficit, visuospatial impairment, psychiatric dysfunction, in the context of normal language abilities, normal extrapyramidal function and normal procedural memory, according to original Filley’s description.^1^ Notwithstanding that some of these features have been used to distinguish white matter dementia from subcortical dementia, we do not agree with all of these, since extrapyramidal signs can also appear in white matter dementia in the context of primarily vascular disease (secondary parkinsonism), as observed in case 1 (CADASIL), or in the context of normopressive hydrocefalus. By the same token, case 3 (PML) presented with expressive dysphasia, a feature often related with cortical dementia. In other words, white matter dementia does not necessarily always have the same clinical profile, since the clinical picture may depend on the specific topography of white matter lesions in the brain. Thus, lesions in the prefrontal area and anterior temporal lobes may produce personality changes and behavioral alterations whereas limbic and other temporal lesions can produce psychotic symptoms^12^ and memory deficits. Posterior white matter lesions can evoke visuospatial deficits and so forth, paralleling occurrences with symptomatological organization in cortical dementias.

The pattern of neuropsychological performance in the presented cases, for instance, can be associated to a general slowing of cognition, i.e., impaired speed of information processing. Psychiatric phenomena (personality changes, apathy, disinhibition, delusions) may also be a syndromic hallmark compounding the clinical picture of this sort of dementia, in the sense that they may be much more common, for example, than in cortical dementia. Further studies specifically addressing this issue must be conducted in a bid to provide evidence for these theories.

In a general sense, whereas the gray matter of the brain caters to information processing, white matter provides for information transfer. In the presence of damaged white matter, information processing occurs only in a slowed and inefficient manner, where there may be no processing at all if the white matter is severely impaired.^2^ This may explain why some of our patients had such severe neuropsychological and behavioral impairment (probably those with structural rather than functional white matter disease) while others did not. Case 4 for instance, with a reversible hydrocephalus, seemed to exhibit less psychiatric symptoms (as demonstrated by the lowest NPI score among the group) and less intense cognitive deficits. In fact, case 4 had almost all his symptoms reversed with a ventricular shunt. Cerebral white matter pathology may not be as complete or permanent as is usually the case with primary neuronal disorders. Given this, an important clinical feature of white matter disorders is that the potential for recovery may be greater than in grey matter disorders.

Even restricted to the frontal lobes, some white matter disorders can produce extensive cognitive dysfunction, as presented in case 2. An anatomical feature of cerebral white matter can probably explain this phenomenon. The frontal lobe white matter establishes a unique relationship between the frontal lobes and more posterior regions of the brain.^4^ In addition to extensive subcortical connections, the frontal lobes are reciprocally connected to the parietal, temporal and occipital lobes by the arcuate (superior longitudinal) fasciculi.

The high prevalence of psychiatric symptoms, insight impairment and attentional deficits in our group could indicate greater involvement of right frontal circuits in the manifestation of this group of features. Perhaps this can be explained by the fact that the ratio of white to gray matter is significantly higher in the right than the left hemisphere, particularly in the frontal lobes.^30^ These observations suggest that diffuse white matter pathology would tend to disrupt attentional systems, frontal lobe function, visuospatial skills, and emotional status.

There are important differences between cortical dementia, subcortical dementia and the concept of white matter dementia. In essence, with regard to memory deficit, there is an encoding deficit in declarative memory for cortical dementia but a retrieval deficit in the sub-cortical and white matter dementias. The difference between sub-cortical and white matter dementia occurs with procedural memory, which is impaired in subcortical dementia, but normal in white matter dementia as it is in cortical dementia. Thus, white matter dementia is seen as a memory retrieval deficit with normal procedural memory.^31^ Another important characteristic that contrasts white-matter dementias with cortical types is the salient presence of neurological features in the former, not only extrapyramidal as in subcortical dementia, but also pyramidal symptoms, urinary incontinence and gait abnormalities.

The composition of marked psychiatric symptoms and personality changes (mainly apathy, but also disinhibition) with neurological signs (pyramidal alone or associated with extrapyramidal signs, ataxia and urinary incontinence) and with specific cognitive impairment (mentioned above), strongly suggests the possibility of a white-matter dementia, rather than a cortical or subcortical form of dementia. It is important to reinforce the notion that, in clinical terms, white matter dementia may exhibit a nonspecific clinical profile and, in neuropathological terms, may present some overlap between white matter disorders and those primarily involving gray matter, which in turn reflect in clinical presentation. As observed with our cases, conventional neuroimaging has enabled more accurate identification of white matter regions participating in neurobehavioral operations, and newer imaging techniques may define white matter connectivity within and between the hemispheres.

## Conclusion

Many different causes of dementia and cognitive impairment may be related to white matter pathology. Cognitive profile of white-matter dementia is characterized by sustained attention deficit, executive dysfunction, memory (mainly retrieval) deficits, insight impairment and occasionally, visuospatial impairment. Behavioral profile is characterized chiefly by frequent and severe psychiatric symptoms especially related to the domains of delusion, agitation, apathy, disinhibition and aberrant motor behavior. Patients with white matter dementia scored highly on the NPI irrespective of the subjacent cause of their dementia.

An appreciation of the neurobehavioral importance of white matter disorders can be of great benefit for patients seen by neurologists and psychiatrists alike, especially because early recognition and treatment can often have an important influence on outcome. A focus on white matter and its disorders has heuristic value and promises to expand knowledge of the brain as an extraordinarily complex organ in which the connectivity provided by white matter is central to cognition, emotion, and consciousness itself. As the details of white matter structure and function become clarified, a more complete picture of the organ of the mind can be expected. The term white matter dementia is intended to alert clinicians and researchers to these issues in the context of white matter and its many disorders.

No external support was sought or received towards this research.

## Figures and Tables

**Figure 4:**
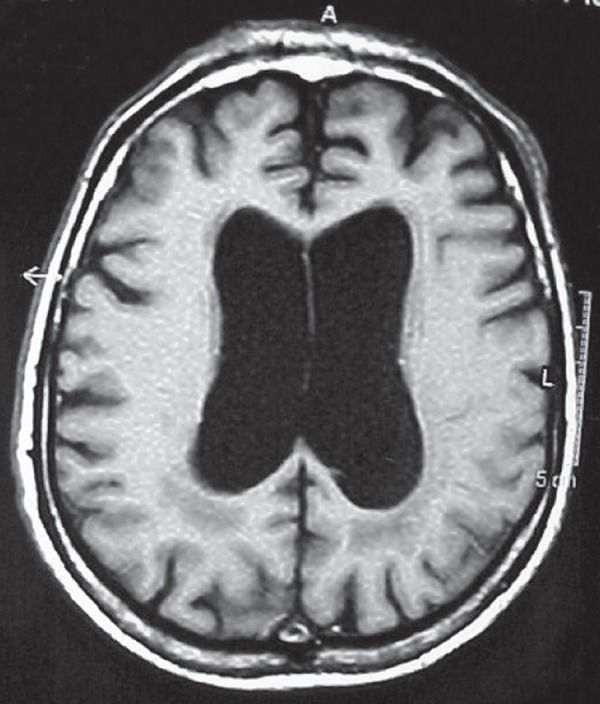
Case 4 – RM (T1 axial) showing hy drocephalus and hypointense white matter (periventricular) foci (mainly posterior).
